# In the genetics of the beholder: gene-environment interplay for internalising and externalising behaviours using polygenic scores and adolescent perceptions of parenting

**DOI:** 10.1007/s00787-025-02804-8

**Published:** 2025-06-30

**Authors:** Nisa R. Rainy, Emma Meaburn, Bonamy R. Oliver, Marthe de Roo, Tina Kretschmer

**Affiliations:** 1 Centre for Brain and Cognitive Development, Birkbeck, University of London, 33 Torrington Square, London, WC1E 7JL UK; 2 Department of Psychology and Human Development, UCL Institute of Education, University College London, London, UK; 3 Faculty of Behavioural and Social Sciences, University of Groningen, Groningen, Netherlands; 4 Department of Psychology, Friedrich-Alexander University Erlangen-Nuremberg, Erlangen, Germany

**Keywords:** Internalising, Externalising, Intergenerational transmission, Genetic nurture, Polygenic score, Psychopathology

## Abstract

Adolescent internalising and externalising behaviours arise from a complex interplay of genetic and environmental factors. Understanding how parents’ genes affect risk for psychopathology in offspring—whether through direct genetic transmission or environmentally mediated genetic nurture—is crucial for identifying causal mechanisms and intervention targets. However, few studies have examined this interplay rigorously in relation to adolescent psychopathology, and findings to date have remained mixed. To tackle this gap in research, we examined both genetic transmission and genetic nurture pathways using polygenic risk for psychopathology on adolescent internalising and externalising behaviours.

The sample comprised 762 genotyped parent-offspring trios from the TRacking Adolescents’ Individual Lives Survey (TRAILS). Parent and offspring polygenic scores for genetic liability to general psychopathology (p-PGS) were computed and jointly modelled to estimate genetically transmitted and genetic nurture contributions to parental reports of offspring internalising and externalising behaviours at age 11.

Our findings indicate that parental polygenic risk for genetic liability to general psychopathology (captured as parental p-PGS) was associated with adolescent internalising—but not externalising—behaviours. Primarily this association was via environmental rather than direct genetic pathways, providing support for genetic nurture (*b* = 0.13, 95% CI = 0.05/0.22, *q-*value = 0.004). However, we found no association between parental p-PGS and adolescent-reported parenting behaviours, limiting our ability to assess whether genetic nurture effects for internalising problems were mediated by adolescent perspectives of parenting.

The study highlights the importance of genetic nurture’s influence on internalising behaviours, and encourages the consideration of genetic influences on perceptions of environmental influences that may be key.

**Supplementary Information:**

The online version contains supplementary material available at 10.1007/s00787-025-02804-8.

## Introduction

Adolescence is a key period for the onset of psychopathology [1]. Compared to other stages of life, susceptibility to psychiatric disorders is greatest during this time with disorders emerging in adolescence often being more persistent and severe [2]. Behaviours may be broadly categorised into internalising and externalising [3]. Internalising behaviours involve emotions directed inward, such as anxiety, social withdrawal, and depression, while externalising behaviours are directed outward, involving interactions with the social environment, such as aggression, impulsivity, and conduct problems [4]. Early presence of these behaviours may predict later psychopathology: early internalising behaviours have been shown to precede anxiety, mood disorders, and suicidality in adulthood [5], while early externalising behaviours predict later substance abuse and criminal behaviour, linked to unstable employment and challenges in social relationships [6]. Understanding the risk factors for psychopathology during adolescence is essential for developing timely and effective interventions to mitigate their pervasive impact on this broad array of outcomes.

Adolescent internalising and externalising behaviours show significant heritability, with twin studies estimating that genetic factors account for 40–50% of variance in internalising [7, 8] and up to 80% in externalising behaviours [9, 10]. Nevertheless, extensive research underscores the importance of diverse environmental factors in adolescent psychopathology, including parental mental health issues, negative parenting strategies, and low parental involvement [11, 12]. Notably, findings from twin and adoption studies suggest that many of these ostensibly ‘environmental’ factors are themselves under genetic influence. For example, parenting behaviours are heritable, implying that parental genes may contribute to child outcomes not only via direct genetic inheritance, but also indirectly via the home environment [13–16].

**Fig. 1:**
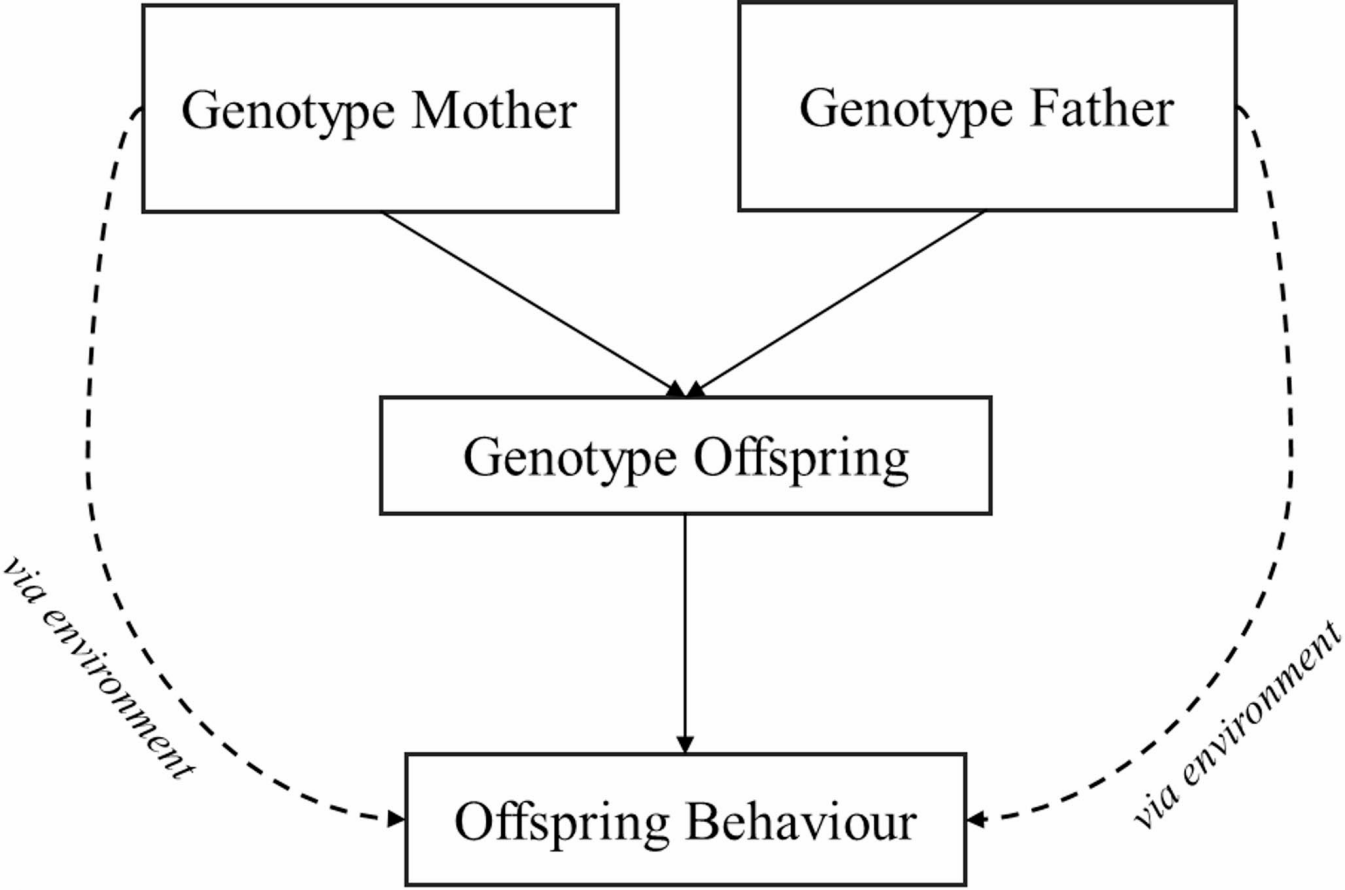
Conceptual framework linking parental genotype and offspring behaviour. Dashed lines indicate the genetic nurture effect (i.e., the effect of parental genotype on offspring behaviour via environmental paths). Solid lines indicate the effect of parental genotype on offspring behaviour via genetic transmission. Jointly modelling parental and offspring PGSs, offspring behaviours and family environment data in this framework allows us to examine (i) if parental PGSs associate with offspring behaviour(s) through environmental transmission accounting for confounding effects of offspring PGS (i.e., genetic nurture), (ii) if parental PGSs associate with offspring behaviour through genetic transmission accounting for environmental confounding (i.e., genetic transmission), and iii) if offspring PGS associates with offspring behaviour accounting for parental PGSs (i.e., child direct genetic effects) [19]

Indeed, previous research on gene-environment interplay suggests that intergenerational associations between parental or family characteristics and adolescent internalising and externalising behaviours may involve both ‘direct’ genetic transmission (i.e., parents pass on genetic predispositions to psychopathology, which in turn contribute to offspring behaviours) and ‘indirect’ genetic effects, whereby the genomes of others, such as parents, influence offspring psychopathology via environmentally mediated pathways—a phenomenon referred to as ‘genetic nurture’ [17]. As the term implies, it is hypothesised that the parental genome shapes nurturing behaviours—such as emotional support, caregiving, and other forms of parenting practices—which in turn influence offspring behaviour. See Fig. 1 [18]. The growing availability of cohorts with behavioural data and polygenic scores (PGSs) for trios (both parents and their biological offspring) provides new opportunities to disentangle genetic transmission from genetic nurture effects within families.

Evidence for genetic nurture has been most robustly established for educational attainment [20], with a landmark study demonstrating that a parental PGS for years spent in education predicted offspring educational outcomes—despite the PGS being composed of alleles not transmitted to the offspring [18]. In contrast, only a limited number of studies have explored genetic nurture effects for adolescent externalising and internalising behaviours using PGSs. For example, a trio PGS study of a population-based Norwegian cohort examined genetic pathways for conduct problems at age 14 using 13 PGSs spanning psychiatric and related conditions, and reported no evidence of genetic nurture [21]. Similarly, PGS studies of ADHD in UK [22] and Dutch [23] adolescent cohorts also found no evidence of such effects. In contrast, genetic nurture was recently reported in a high-risk sample for alcohol use disorders, where parental externalising behaviours mediated genetic nurture effects for adolescent externalising behaviours [24]. To date, only one published study has explored genetic nurture effects on adolescent internalising behaviours [25]. This study found no association between adolescent emotional problems and PGSs for psychiatric and neurodevelopmental conditions in a European genetic ancestry sample, but reported weak evidence of genetic nurture effects in a South Asian ancestry sample.

Notably, the studies described above employed PGSs for single diagnoses (e.g., ADHD, conduct disorder, and anxiety disorder) or broader clusters of behaviours (e.g., internalising and externalising problems). This approach treats psychiatric disorders or symptoms as genetically distinct, yet converging evidence points to substantial genetic overlap across diagnostic categories. Large-scale multi-variate GWASs have revealed substantial genetic correlations among psychiatric conditions, which converge into a latent transdiagnostic ‘genomic p’ factor that captures shared genetic liability to psychopathology [26, 27]. Genomic p is stable across development and correlated with phenotypic ‘p’ in a UK cohort [28].

In this study, we utilised trio data from the TRacking Adolescents’ Individual Lives Survey (TRAILS) cohort to better understand how psychopathology persists within families by investigating the transmission of parental genetic liability to psychopathology—indexed by a PGS for genomic p—to offspring behaviours during adolescence. We used a genomic p PGS (p-PGS) derived from Genomic Structural Equation Modelling (Genomic SEM) applied to the latest well-powered GWAS summary statistics for 11 psychiatric disorders [29], which obviated the need to develop and validate a new p-PGS in our (smaller) cohort. The p-PGS offers two key advantages for our study. First, conceptually, it aligns with our aim to test intergenerational transmission of broad genetic risk for adolescent internalising and externalising behaviours, rather than for any one specific diagnosis. Second, empirically, the p-PGS has been shown to outperform many disorder specific polygenic scores in predicting childhood emotional and behavioural difficulties [28, 30], making it an ideal candidate for examining genetic nurture effects.

We tested for genetic transmission, child direct genetic effects, and genetic nurture effects linking the p-PGS to dimensional measures of parent-reported offspring internalising and externalising behaviours. When environmental data, such as parenting, are also available, it becomes possible to test specific environmental processes that may mediate genetic nurture effects. The parent-offspring relationship is one of the most salient processes for internalising and externalising behaviours throughout childhood and adolescence [11, 31–34]. Hence, where genetic nurture effects were detected, we examined whether parenting practices mediated the association.

We focused on adolescent reports of the parenting they experience, due to the important nature of perceptions of these experiences [35]. Importantly, perceptions of the home environment can differ considerably. In particular, parent and offspring agreement about parenting is moderate at best, and the heritability of parenting is higher when reported by parents than when reported by offspring, suggesting that our understanding of gene-environment interplay may be keenly dependent on the perceiver. Here, we present the first study to examine genetic nurture using parent-reported child behaviours and child-reported parenting to address potential confounding by genetically-influenced reporter bias. By incorporating parenting constructs within genetic nurture pathways, we aim to identify modifiable targets for intervention and support strategies that may help mitigate internalising and externalising behaviours.

## Methods

### Preregistration

The study methods and hypotheses were pre-registered on the Open Science Framework (https://osf.io/wxv6r). Analyses not preregistered are indicated.

### Sample

The data used here come from the TRacking Adolescents’ Individual Lives Survey (TRAILS), a prospective longitudinal cohort study of Dutch adolescents who have been followed bi- or tri-annually from 11 (wave 1) to age 32 (wave 8) [36]. The current study uses phenotype data only from wave 1 and genetic data from wave 3. Wave 1 is the only wave where both parent-reported internalising and externalising behaviours and child-reported parenting were available, allowing us to model mediation.

The TRAILS study includes two samples: a general population sample and a sample of children who had been in contact with mental health services. The general population sample was recruited from five municipalities in the northern Netherlands, covering urban and rural areas. Initially, 135 primary schools were approached, with 122 participating. Out of 2,935 invited children, 2,229 (51% female, as indicated by participants during the first wave) participated at wave 1 in 2001–2002, with a mean age of 11.1 years. The second sample comprises 543 children identified through referral to child and adolescent mental health services (CAMHS) in the northern Netherlands prior to age 11. Inclusion was based solely on record of this contact; no specific diagnosis was required, nor was a mental health assessment conducted as part of this sample’s recruitment. Detailed TRAILS cohort information, including attrition, is available in prior publications [36, 37], and descriptive statistics are presented in Supplementary Table S1.

Parental mental health data were collected at wave 3 (when children were approximately 16 years old). Both parents were invited to complete a questionnaire and interview that assessed psychopathology using vignette-style items developed by the TRAILS team. These vignettes described symptoms of depression, anxiety, addiction, antisocial behaviour, and psychosis. Descriptive statistics are provided in Supplementary Table S2.

### Measures

#### Polygenic score for genomic p (p-PGS)

PGS was calculated as the weighted sum of an individuals’ alleles associated with a specific trait, providing an estimate of an individual’s genetic predisposition to that trait relative to other individuals in the population [38]. For the present study, a PGS for genomic p (p-PGS) was calculated for 1,694 TRAILS participants, including 762 complete trios, using LDPred2-auto [39], which automatically estimates SNP heritability and the proportion of causal variants from the data, hence removing the need for a validation dataset. PGSs for 10 psychiatric disorders were also computed (not preregistered) to explore the extent to which the p-PGS is correlated with disorder-specific PGSs in the TRAILS cohort. Information on the genotyping of the TRAILS sample is provided elsewhere [40].

For the p-PGS, effect-size weights were derived from a multivariate GWAS analysis of 11 psychiatric disorders using GWAS summary statistics and Genomic SEM. This p-GWAS incorporated the most recent publicly available datasets for anxiety disorder [41], major depressive disorder [42], posttraumatic stress disorder [43], bipolar disorder [44], schizophrenia [45], attention-deficit hyperactivity disorder [46], autism spectrum disorder [47], anorexia nervosa [48], obsessive-compulsive disorder [49], problematic alcohol use [50], and Tourette syndrome [51]. Genomic SEM first uses multivariable LD score regression to estimate the genetic covariance matrix and sampling covariance matrix across datasets, and then fits a structural model to identify a latent transdiagnostic genomic factor (i.e., genomic p) that captures shared genetic liability. Full methodological details are available in Keser et al. (2024) [29].

There was no overlap in participants between TRAILS and GWAS summary datasets used by Keser et al. (2024) to generate the genomic p GWAS summary statistics. All summary statistics used are based on samples of European ancestry only, and only individuals of European genetic ancestry were included in our analyses. We controlled for population stratification by the regressing each PGS on the first 20 genomic ancestry principal components (PCs) and used residuals in all analyses.

### Parental report of offspring internalising and externalising behaviours

Parent reports of offspring internalising and externalising behaviours were collected from parents over a 6-month period when the offspring were 11 years old using the Child Behaviour Checklist (CBCL) [4]. The CBCL consists of 118 items on various aspects of child psychopathology, including 32 items for internalising behaviours and 30 items for externalising behaviours [52]. Each CBCL item was rated on a three-point scale (0 = *not true*, 1 = *somewhat or sometimes true*, 2 = *very or often true*). Previous research reported robust psychometric properties for the CBCL, with high internal consistency (average *α* = 0.92) for internalising and externalising behaviours [53].

### Child report of parenting behaviours

Child perceptions of parenting were assessed at age 11 using the Egna Minnen Beträffande Uppfostran (Swedish for “Memories of my upbringing”) for Children (EMBU-C) [54, 55]. The questionnaire comprises 47 items asked separately for both father and mother that form three parenting scales: Warmth (18 items, e.g., “Does your mother/father make it obvious that she/he loves you?”), Rejection (17 items, e.g., “Is your mother/father sometimes harsh and unkind to you?”), and Overprotection (12 items, e.g., “ Do you think that your parents are too worried about things happening to you?”). Responses were rated on a four-point scale (ranging from 1 = *never* to 4 = *almost always*). Internal consistency was good to excellent for this sample (*α* warmth father = 0.89; *α* warmth mother = 0.87; *α* rejection father = 0.84; *α* rejection mother = 0.83; *α* overprotection father = 0.64; *α* overprotection mother = 0.67).

### Analytic procedures

We used Stata [56] to compute pairwise correlations between parents’ and offspring’s p-PGSs, offspring internalising and externalising behaviours, and parenting practices (warmth, rejection, overprotection). We also computed pairwise correlations between 10 psychiatric disorder PGSs, p-PGS, and offspring internalising and externalising behaviours (not preregistered). Path models were estimated in Mplus version 8.6 [57], including the mediation analysis of genetic nurture effects using the ‘Model Indirect’ command. Model fit parameters were computed using maximum likelihood estimation and robust standard errors.

### Path analysis

**Fig. 2:**
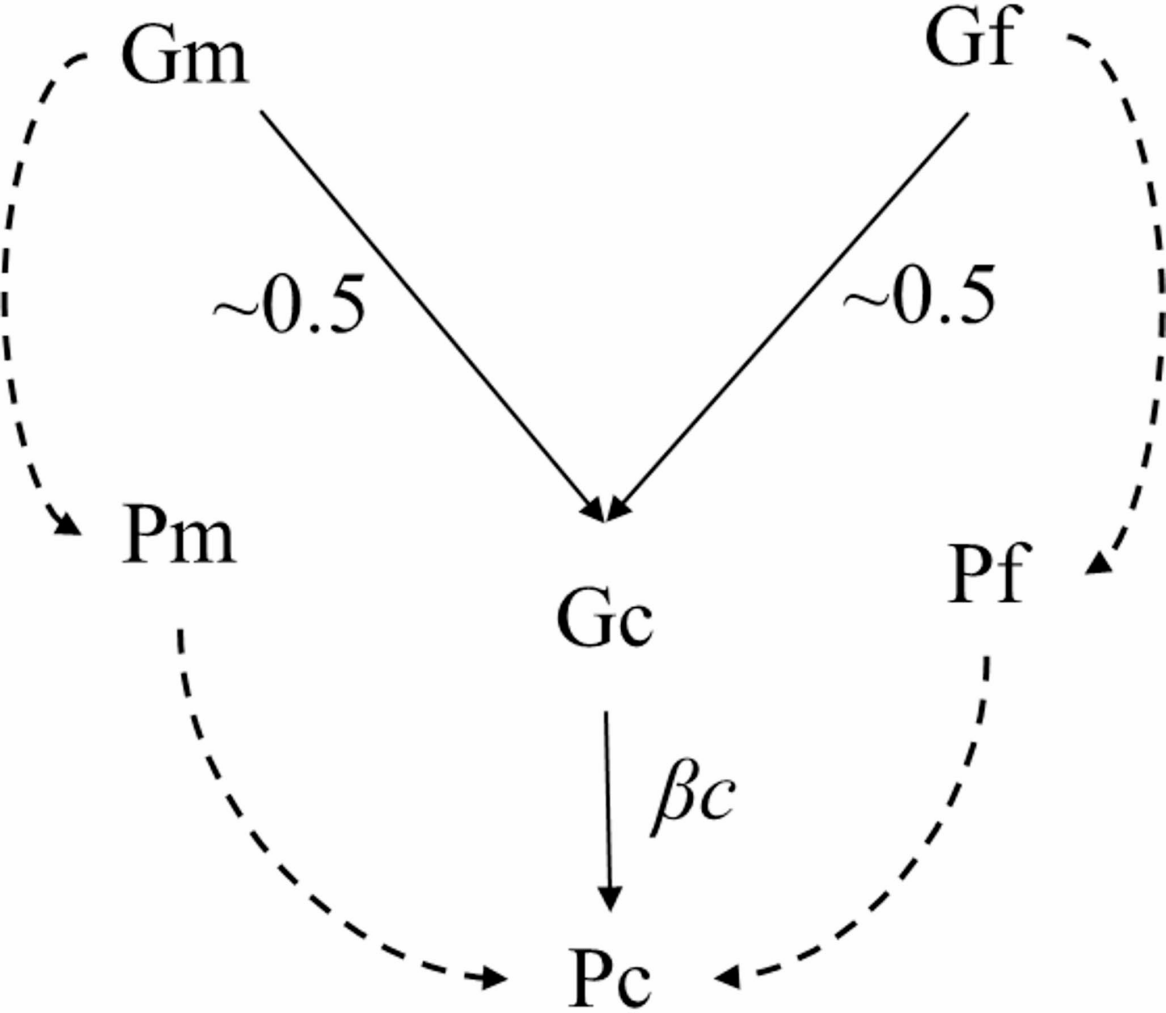
Associations between parental p-PGSs and offspring internalising and externalising. *G* = genotype, *P* = phenotype, *f* = father, *m* = mother, *c* = child. Genetic transmission effects reflect the pathway through the solid lines *(G*_*f/m*_*→ G*_*c*_*→ P*_*c*_*)*, where *G*_*c*_*→ P*_*c*_ is the child’s direct genetic effect, estimated as 0.5 × *β*_*c*_. Genetic nurture effects are represented through the dashed lines (*G*_*f/m*_*→ P*_*f/m*_*→ P*_*c*_). If associations between parental genotype *(G*_*m*_*or G*_*f*_*)* and offspring phenotype *(P*_*C*_*)* remain after controlling for offspring genotype *(G*_*c*_*)*, this suggests genetic nurture effects. Environmental mediation of genetic nurture effects are represented by parental phenotype *(P*_*m*_*or P*_*f*_ ). Child direct genetic effects are indicated by the solid line (*Gc → Pc*) and represent the effect of the offspring’s own genotype on the offspring’s trait, independent of parental genotype

We evaluated path models for internalising and externalising behaviours, with each model examining genetic transmission, genetic nurture, and child direct genetic effects (Fig. 2). Genetic transmission was estimated as the effect of parental p-PGSs on offspring internalising and externalising behaviours through the offspring’s own p-PGS. Genetic nurture effects were determined as the association between parental p-PGSs and offspring internalising and externalising behaviours after accounting for the offspring’s p-PGS. Child direct effects were estimated as the effects of offspring’s own p-PGS on internalising and externalising behaviours, independent of parental p-PGSs. *P-*values of genetic nurture were adjusted for multiple testing across all tests by applying the false discovery rate (FDR) correction (i.e., N traits x N effects). Specifically, *p*-values for genetic nurture were corrected for 4 tests: 2 traits (internalising and externalising behaviour) and 2 effects, corresponding to each parent. If genetic nurture effects were found with *q*-values (FDR corrected p-values) < 0.05, mediating parenting measures were added to the model.

We used the Model Indirect command in Mplus to estimate the effects of parents’ p-PGSs on offspring externalising and internalising behaviours via offspring’s own p-PGS (e.g., genetic transmission) and via parenting behaviour (e.g., the mediation path of genetic nurture). We anticipated genetic relatedness between parents and their offspring (*Gm/Gf →Gc*) of ~ 0.5. See Fig. 2. Full information maximum likelihood estimation was used to handle missing phenotypic data, utilising all available data. All analyses were controlled for age at testing, sex, and socioeconomic status. Genomic ancestry PCs were regressed out during PGS construction, and the resulting residualised scores were used in the path models; PCs were therefore not included as covariates in the path models. We report standardised estimates and 95% confidence intervals for path estimates.

### Sensitivity analyses

Given the moderate phenotypic and genetic correlations between internalising and externalising behaviours, all analyses were repeated while controlling for the respective other trait (i.e., controlling for externalising behaviours in internalising models and vice versa). See supplementary material for more details.

## Results

### Validation step

We first validated the p-PGS in the TRAILS cohort by testing its association with offspring externalising and internalising behaviours (see Table 1). As expected, offspring p-PGS was associated with both externalising (*r* =.12, *p* <.001) and internalising behaviours (*r* =.10, *p* <.001). Both mother (*r* =.10, *p* =.001) and father (*r* =.10, *p* =.006) p-PGSs were correlated with parental report of offspring externalising behaviours. Only mother p-PGS (*r* =.14, *p* <.001) was significantly correlated with parental report of offspring internalising behaviours. The significant correlation between mother and father p-PGSs (*r* =.08, *p* =.029) suggest assortative mating for genomic liability to psychopathology. The p-PGS was positively correlated with all disorder-specific PGSs. It also showed stronger correlations with both internalising and externalising behaviours than any disorder-specific PGS—except the PGS for ADHD, which had a higher correlation with externalising (*r* =.16, *p* <.001). See Supplementary Figure S1 and Table S3.

No significant correlations were found between parents’ p-PGSs and offspring reports of their respective parenting practices. However, offspring p-PGS was significantly correlated with their perceptions of parental overprotection (mother: *r* =.07, *p* =.003; father: *r* =.06, *p* =.009). As expected, both offspring externalising and internalising behaviours were positively correlated with parental rejection and overprotection, and negatively correlated to parental warmth.

We found gene-environment interplay, where offspring p-PGS was associated with both parent-reported offspring behaviour problems and child-reported parental overprotection (mother: *r* =.07, *p* =.003; father: *r* =.06, *p* =.009), which may be suggestive of evocative gene–environment correlation (rGE). To test the evocative rGE more directly, we regressed the parental overprotection on offspring p-PGS, while adjusting for parental p-PGSs (not preregistered). However, once shared genetic liability was accounted for, the associations between offspring p-PGS and parental overprotection were no longer significant (mother: *β* = − 0.008, *p* =.857; father: *β* = − 0.029, *p* =.539).

All path models demonstrated good fit, as evidenced by the comparative fit index (CFI = 0.94–0.95), Tucker-Lewis index (TLI = 0.90–0.94), standardised mean square residual (SMSR = 0.04), and root mean square error of approximation (RMSEA = 0.03–0.04) (see Supplementary Table S4).

### Genetic transmission and genetic nurture effects for externalising behaviours

**Fig. 3:**
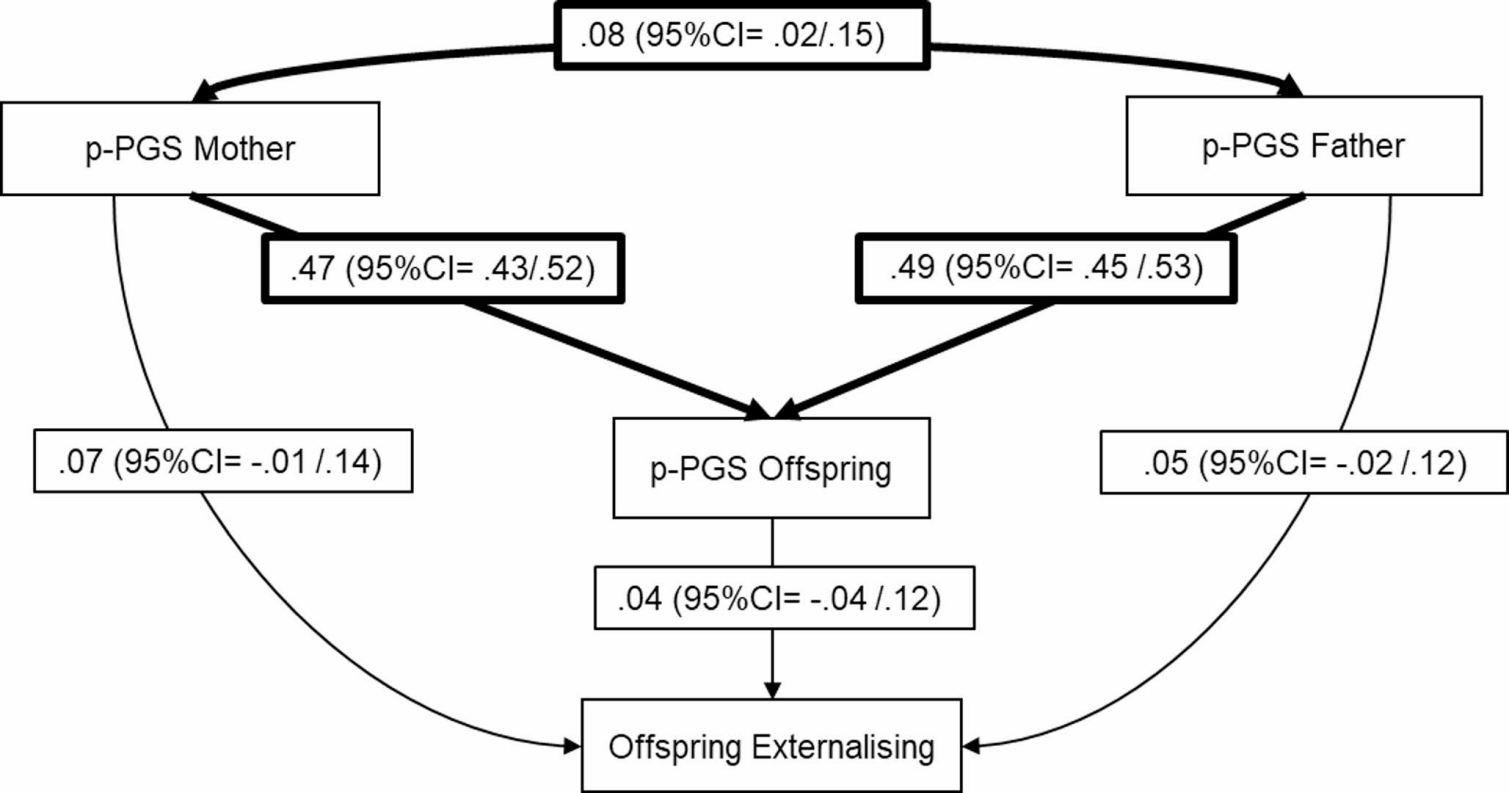
Path model for externalising behaviour (*N* = 2603). The model is just-identified. Reported estimates are standardised with significant estimates shown in bold arrows. Significant assortative mating is observed in the model. Polygenic scores were regressed on the first 20 principal components; residuals were used in this analysis. Offspring externalising was adjusted for age, sex, and socioeconomic status

As expected, we observed significant genetic relatedness between parents and offspring (father: *b* = 0.47, 95% CI = 0.43/0.52; mother: *b* = 0.49, 95%CI = 0.45/0.53) (See Fig. 3). However, after accounting for parental p-PGSs, the direct effect of offspring’s p-PGS on externalising was small and non-significant (*b* = 0.04, 95% CI = − 0.04 /0.12), resulting in no clear path for genetic transmission (*b* = 0.02, 95% CI = − 0.02/0.06). We did not find evidence of genetic nurture as parents’ p-PGSs did not predict externalising after controlling for the offspring’s p-PGS (p-PGS father: *b* = 0.07, 95% CI = − 0.01 /0.14, *q-*value = 0.169; p-PGS mother: *b =* 0.05, 95% CI = − 0.02 /0.12, *q-*value = 0.169).

**Table 1:** Bivariate correlations between trio p-PGSs and internalising and externalising behaviours

	1	2	3	4	5	6	7	8	9	10	11	12	13
1. p-PGS C	1												
2. p-PGS M	0.53 (< 0.001)	1											
3. p-PGS F	0.53 (< 0.001)	0.08 (0.029)	1										
4. INT	0.10 (< 0.001)	0.14 (< 0.001)	0.06 (0.103)	1									
5. EXT	0.12 (< 0.001)	0.10 (< 0.001)	0.10 (0.005)	0.54 (< 0.001)	1								
6. WAR M	− 0.01 (0.833)	0.01 (0.861)	0.02 (0.659)	− 0.05 (0.019)	− 0.15 (< 0.001)	1							
7. REJ M	0.00 (0.918)	0.01 (0.696)	0.05 (0.177)	0.09 (< 0.001)	0.25 (< 0.001)	− 0.33 (< 0.001)	1						
8. OVP M	0.07 (0.003)	0.06 (0.073)	0.04 (0.193)	0.07 (< 0.001)	0.12 (< 0.001)	0.18 (< 0.001)	0.43 (< 0.001)	1					
9. WAR F	− 0.04 (0.161)	− 0.04 (0.189)	0.01 (0.832)	− 0.10 (< 0.001)	− 0.17 (< 0.001)	0.77 (< 0.001)	− 0.21 (< 0.001)	0.12 (< 0.001)	1				
10. REJ F	0.03 (236)	0.04 (0.241)	0.05 (0.180)	0.14 (< 0.001)	0.26 (< 0.001)	− 0.19 (< 0.001)	0.65 (< 0.001)	0.37 (< 0.001)	− 0.35 (< 0.001)	1			
11. OVP F	0.06 (0.009)	0.06 (0.038)	0.04 (0.208)	0.09 (< 0.001)	0.14 (< 0.001)	0.19 (< 0.001)	0.34 (< 0.001)	0.81 (< 0.001)	0.22 (< 0.001)	0.40 (< 0.001)	1		
12. Age	0.05 (0.046)	0.02 (0.544)	0.02 (0.486)	− 0.03 (0.156)	0.013 (0.506)	− 0.15 (0.426)	− 0.01 (0.442)	0.01 (682)	0.00 (0.853)	− 0.03 (0.112)	− 0.03 (0.112)	1	
13. Sex	− 0.04 (0.099)	0.02 (0.546)	0.04 (0.304)	0.03 (0.124)	0.19 (< 0.001)	− 0.12 (< 0.001)	0.10 (< 0.001)	0.04 (0.043)	− 0.11 (< 0.001)	0.12 (< 0.001)	0.04 (0.019)	0.03 (0.071)	1

Note: p-PGS = Polygenic score for genomic p, C = Child, M = Mother, F = Father, INT = Internalising behaviours, EXT = Externalising behaviours, WAR = Warmth, REJ = Rejection, OVP = Overprotection

### Genetic transmission and genetic nurture effects for internalising behaviour

**Fig. 4:**
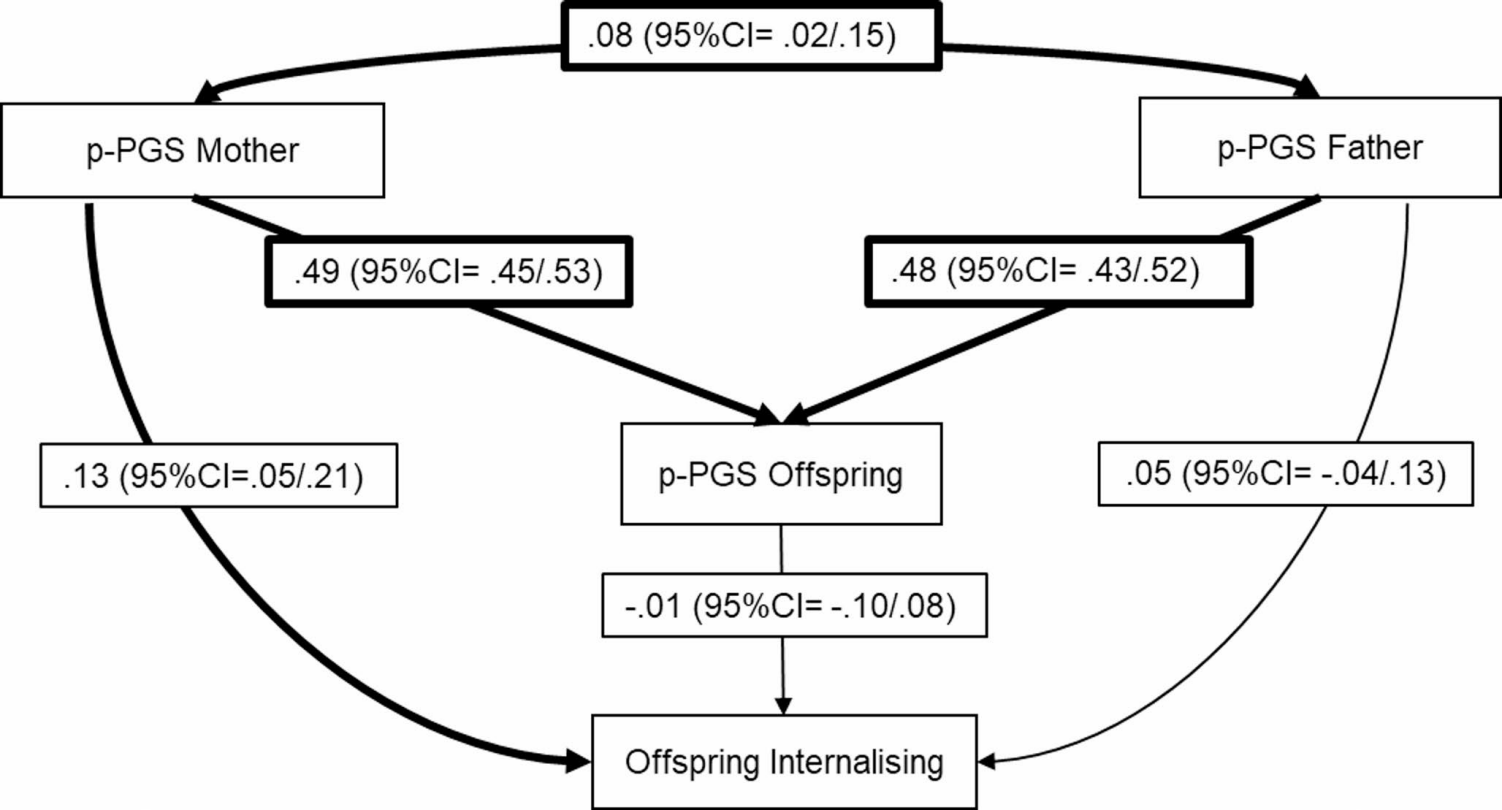
Path model testing genetic nurture for internalising behaviour (*N* = 2598). The model is just-identified. Reported estimates are standardised with significant estimates shown in bold arrows. Significant assortative mating is observed in the model. Polygenic scores were regressed on the first 20 principal components; residuals were used in this analysis. Offspring externalising was adjusted for age, sex, and socioeconomic status

We observed a similar pattern of findings for internalising behaviours at age 11 with significant genetic relatedness between parents and offspring (father: *b* = 0.48, 95% CI = 0.43/0.52; mother: *b* = 0.49, 95% CI = 0.45/0.53), but diminished and non-significant estimates of offspring’s direct effect (*b* = − 0.01, 95% CI = − 0.10/0.08) (see Fig. 4). No significant genetic transmission was observed (*b* = − 0.004, 95% CI = − 0.05/0.04). However, in contrast with externalising behaviours, we found evidence of genetic nurture via the mother’s p-PGS (*b* = 0.13, 95% CI = 0.05/0.22, *q-*value = 0.004) but not via the father’s (*b* = 0.04, 95% CI = − 0.05/0.13, *q-*value = 0.291). Given the validation step showed no significant links from parent p-PGSs to parenting behaviour and offspring outcomes, planned exploration of potential mediators in the genetic nurture pathway was not conducted.

**Sensitivity Analysis.** Sensitivity analysis was performed by re-analysing the path models with internalising and externalising outcomes allowed to covary. The results were consistent with the main analysis. (see Supplementary Figures S2–S3).

## Discussion

This study examined the direct genetic and indirect environmental pathways through which polygenic liability for psychopathology on internalising and externalising behaviours is transmitted from parents to offspring. Using trio genomic data, parent-reported offspring outcomes, and child-reported perspectives of parenting, we found that parental genetic predisposition for psychopathology influences adolescents’ internalising behaviours through environmental rather than direct genetic pathways—beyond the effect of shared genes.

Different to our expectations, we found no evidence that adolescents’ own genetic makeup directly influenced their internalising or externalising behaviours (i.e., child direct genetic effect). While the initial pairwise correlations showed robust associations between offspring PGS for genomic p and parent-reported behaviours of psychopathology, these associations became non-significant when parental p-PGSs were accounted for in the model.

One possibility for the absence of significant direct genetic effects is that, once environmental pathways are accounted for, genetic transmission effects may become attenuated, as they could operate through a more complex gene-environment interplay. Situational familial characteristics—such as socioeconomic status, spousal relationships, and parental social support—are candidate environmental pathways that we did not examine in this study but have been reported to moderate intergenerational transmission of psychopathology [58]. These factors may create environmental conditions that can ‘override’ the effects of a child’s alleles on their own behaviour. In other words, instead of directly observing the child’s genetic contribution to certain traits, what we see might be more strongly shaped by environmental influences. However, alternative explanations include the limited predictive power of the p-PGS, and potential suppression effects due to overlapping variance between child and parental p-PGSs.

We also observed an intriguing pattern of gene-environment interplay in the pairwise correlations, where offspring genetic predispositions were associated with both parent-reported offspring behaviour problems and child-reported parental overprotection. This may reflect evocative rGE, whereby offspring’s genetically influences behaviours elicit specific parenting responses [15, 59]. However, these associations became non-significant after controlling for parental p-PGSs, suggesting that other mechanisms may be at play. Future research using alternative genetically informed designs—such as sibling comparison or longitudinal models—will be better suited to disentangle this association.

Our findings did not support genetic nurture as a source of variance in adolescent externalising behaviours. This finding aligns with several other studies. For example, a previous study using the same cohort found no evidence of genetic nurture effects on externalising behaviours, nor environmental mediation via family dysfunction [40]. Another Dutch cohort study reported that a non-transmitted PGS for ADHD did not predict ADHD behaviours in 12-year-old offspring [23]. Similarly, a study based on the Norwegian Mother, Father, and Child Cohort Study, which examined 13 PGSs to investigate parental risk factors for conduct disorder in 14-year-old offspring, found no evidence of genetic nurture [21]. Likewise, in a study using a large UK sample, no genetic nurture effects in adolescence were found, as non-transmitted parental alleles for ADHD were not associated with ADHD liability compared to control participants [22]. To date, only one study, conducted in a high-risk sample of individuals with alcohol use disorders, has reported (a small) genetic nurture effect on externalising behaviours at age 15 [24].

A possible explanation for the absence of genetic nurture effects on externalising behaviours here is the timing of behaviour assessment [58]. Genetic nurture effects may be age-dependent, with some effects emerging more strongly earlier in development and others appearing later. Supporting this, studies have detected genetic nurture effects on externalising in both childhood and adulthood, despite their absence in adolescence. In the Norwegian cohort mentioned above, genetic nurture effects for a PGS for polygenic p—akin to the p-PGS used in our study—and for autism were associated with conduct problems and hyperactivity/inattention at age eight [30], and maternal genetic nurture effects for a neuroticism PGS were associated with ADHD traits [19]. In adulthood, genetic nurture effects have been reported for externalising traits, such as aggressive behaviour [60] and ADHD [23] in the Dutch cohort.

In contrast to externalising behaviours, we found evidence for genetic nurture for internalising behaviours for maternal—but not paternal—genetic predisposition, suggesting that these effects may be driven by mothers. This parent gender-specific effect aligns with prior research on intergenerational transmission of psychopathology, which shows maternal, rather than paternal, psychopathology is linked to adolescent internalising behaviours [61, 62], primarily through environmental mechanisms [63]. Recent studies also indicate that maternal genetic nurture can significantly influence physical and mental health [19, 64]. One plausible explanation is that mothers, often the primary caregivers, shape both the offspring’s upbringing and prenatal environment, the latter of which is critical for development and has been suggested as a critical window for genetic nurture effects. That said, offspring behaviours were reported by mothers in the absolute majority of cases (93%), which also raises the possibility of shared method variance playing a part.

Although our finding contrasts with a previous study that found no genetic nurture effects on adolescent internalising behaviours [25], this discrepancy may stem from differences in the genetic measures used. We employed a PGS for the general genetic liability to psychopathology, rather than a domain-specific PGS. In the same cohort, our analysis shows that PGS for genomic p accounts for more variance in externalising behaviour (*R*^*2*^ = 1.44%) than a domain-specific externalising PGS (*R*^*2*^ = 0.49%) used in a prior study [40], suggesting that the general genetic liability measure may serve as a more holistic PGS for studying genetic nurture effects. Supporting this, a recent study on the intergenerational transmission of childhood psychopathology [30], which used a much larger sample, constructed a similar PGS for genomic p, derived from the first principal component of PGSs for eight major psychiatric disorders. Their findings indicated that a PGS for genomic p outperformed disorder-specific PGSs in predicting children’s emotional and behavioural difficulties, and could capture maternal and paternal indirect genetic effects across both general and specific psychopathology domains, with the exception of inattention and anxiety. These findings highlight the value of taking a transdiagnostic approach and serve as a foundation for generating more specific hypotheses.

Our planned mediation analysis could not be performed due to the lack of correlation between parental p-PGSs and parenting measures, despite robust evidence for the heritability of parenting [14]. This absence of association may be due to our reliance on child-reported measures of parenting behaviour. On the one hand, this could be considered to introduce biases relating to the fact that the child’s subjective perception may be reflective of the child’s own genetic predispositions. Supporting this idea, pairwise correlations showed a significant association between offspring p-PGS and their report of parents’ behaviours. Crucially, on the other hand, this notion may be the mirror of what is seen using parent reports of parenting, that is, parent perceptions and reporting reflect their own genetics—which could also be considered “bias”. In other words, genetic nurture mediation may be more evident when parents themselves report on the environments they provide, because their own genetic makeup likely shapes both their behaviours and their perceptions of those behaviours. These possibilities underscore that, while our cross-informant design aimed to minimise shared-method bias, it does not preclude the possibility of genetically-influenced reporter perception effects. We argue that it is also possible that perception effects are not bias—rather that the nature and influence of parenting behaviours for children’s outcomes are complex, and may be partially in the genetics of the beholder. While our findings might be interpreted as indicating a minimal parental role in mediating genetic nurture, they instead highlight the complexity of genetic pathways to parenting.

The strengths of this study include the availability of genetic data capturing general genetic liability to psychopathology from both parents and their offspring, as well as phenotype data of parent-reported offspring behaviour and child-reported parenting—an approach that, to our knowledge, has not been used in previous research. However, several limitations need to be noted. First, our modest sample size limits the statistical power to detect very small genetic nurture effects. For comparison, a similar intergenerational transmission study using a trio PGS design, with a sample size ten times larger (*N* = 15,000), had at least 80% power to detect a small indirect genetic effect (*β* = 0.03) [30]. Given our smaller sample, our ability to detect such effects is likely limited, which may also affect the generalisability of our findings.

Second, our analysis was based on data from a single time point in late childhood, which may not capture effects that emerge over time or later in development. While follow-up data on a variety of topics including psychosocial adjustment, education and work, and social relationships from adulthood are available for this cohort [65], adult outcomes were beyond the scope of the present study and were not included in our analysis. Our aim was to examine proximal correlates of parenting in early adolescence, which may fade or change over time. As such, we did not attempt to model time-dependent associations. Future research could build on this work by linking the genetic nurture pathways explored in our study with later diagnostic or functional outcomes. Longitudinal designs with repeated measures are necessary to better understand how direct genetic effects and indirect genetic effects contribute to variability in psychopathology behaviours over time.

Third, while we used child-reported parenting measures to mitigate self-report bias from parents, these measures reflect the child’s subjective perception, which may not fully represent the environmental influences we intended to capture. Incorporating a range of observational methods, such as cross-rater parenting measures—including those provided by parents themselves—could provide a more nuanced understanding of gene-environment interplay. Fourth, our sample consisted of individuals of European ancestry, which limits the generalisability of our findings to more diverse populations. Lastly, expanding the range of environmental variables measured, such as additional parental factors, would provide much needed insight into the environmental mechanisms by which parental genotype shapes adolescent internalising behaviours. For instance, parental internalising behaviours could mediate this process, as developmental theories of anxiety suggest that children and adolescents may develop anxious behaviours from their parents through modelling [66], and parental criticism—particularly from mothers—has been linked to adolescent internalising behaviours [67].

## Conclusion

Our findings provide evidence for genetic nurture effects in adolescent psychopathology, indicating that parental genetic liability to psychopathology influences internalising —but not externalising—behaviours through environmental rather than genetic transmission pathways. This adds to growing evidence that parental genetic influences can shape early adolescent internalising behaviours through environmental mechanisms, and underscores the value of family-focused intervention strategies designed to mitigate intergenerational risk for psychopathology. Our study also highlights the importance of accounting for reporter effects in genetic nurture research. Future research should incorporate multi-reporter designs and richer environmental measures to better understand the pathways through which parental genotype influences adolescent internalising behaviours.

## Electronic supplementary material

Below is the link to the electronic supplementary material.

Supplementary Material 1

## Data Availability

TRAILS data can be requested through https://www.trails.nl/en/researchers/working-with-trails.
