# Supplementary material for: In the genetics of the beholder: gene-environment interplay for internalising and externalising behaviours using polygenic scores and adolescent perceptions of parenting
Source: Eur Child Adolesc Psychiatry. 2025 Jun 30;34(12):4009–22. doi: 10.1007/s00787-025-02804-8 (PMC12743030; doi:10.1007/s00787-025-02804-8)
Supplement: Supplementary file 1 — Supplementary Material 1 [file 787_2025_2804_MOESM1_ESM.docx]

**Supplementary Files**

Table S1. Descriptive statistics of the genotype and phenotype data.

| **Variable** | ***N*** | **Mean** | ***(SD)*** | **Median** |
| --- | --- | --- | --- | --- |
| p-PGS child | 1676 | 1.76 | 0.22 | 1.76 |
| p-PGS mother | 1087 | 1.74 | 0.21 | 1.75 |
| p-PGS father | 870 | 1.73 | .204 | 1.74 |
| Externalising child | 2551 | 0.29 | 0.24 | 0.23 |
| Internalising child | 2541 | 0.29 | 0.23 | 0.25 |
| Warmth mother | 2695 | 3.27 | 0.50 | 3.39 |
| Rejection mother | 2694 | 1.50 | 0.34 | 1.41 |
| Overprotection mother | 2694 | 1.93 | 0.41 | 1.92 |
| Warmth father | 2631 | 3.12 | 0.58 | 3.22 |
| Rejection father | 2629 | 1.51 | 0.36 | 1.47 |
| Overprotection father | 2630 | 1.79 | 0.40 | 1.75 |

Table S2. Descriptive statistics of parental psychopathology at wave 3

|  | Mother | Father |
| --- | --- | --- |
| Ever had a period of depressive symptoms of at least two weeks | I think so but I'm not sure: 5.61%  Yes: 28.84%  (n=1231) | I think so but I'm not sure: 4.93%  Yes: 18.29%  (n=913) |
| Ever had anxiety symptoms | I think so but I'm not sure: 3.42%  Yes: 18.88%  (n=1229) | I think so but I'm not sure: 3.74%  Yes: 11.21%  (n=910) |
| ASD symptoms | I think so but I'm not sure: 0.57%  Yes: 0.49%  (n=1231) | I think so but I'm not sure: 0.99%  Yes: 0.99%  (n=912) |
| Problems concentrating or being too busy and impulsive | I think so but I'm not sure: 1.46%  Yes: 2.76%  (n=1230) | I think so but I'm not sure: 2.63%  Yes: 3.51%  (n=911) |
| Ever addicted to a substance | I think so but I'm not sure: 3.09%  Yes: 3.33%  (n=1231) | I think so but I'm not sure: 3.62%  Yes: 6.48%  (n=911) |

Table S3. Correlation matrix between p-PGS, disorder specific-PGSs, and internalising and externalising behaviours

|  | 1 | 2 | 3 | 4 | 5 | 6 | 7 | 8 | 9 | 10 | 11 | 12 | 13 |
| --- | --- | --- | --- | --- | --- | --- | --- | --- | --- | --- | --- | --- | --- |
| 1. p-PGS C | 1 |  |  |  |  |  |  |  |  |  |  |  |  |
| 2. PGS ADHD C | .49 (<.001) | 1 |  |  |  |  |  |  |  |  |  |  |  |
| 3. PGS ASD C | .29 (<.001) | .30 (<.001) | 1 |  |  |  |  |  |  |  |  |  |  |
| 4. PGS OCD C | .06 (.018) | -.07 (.003) | -.01 (.665) | 1 |  |  |  |  |  |  |  |  |  |
| 5. PGS SCZ C | .56 (<.001) | .18 (<.001) | .13 (<.001) | .06 (.020) | 1 |  |  |  |  |  |  |  |  |
| 6. PGS BP C | .54 (<.001) | .20 (<.001) | .11 (<.001) | .03 (.195) | .44 (<.001) | 1 |  |  |  |  |  |  |  |
| 7. PGS PTSD C | .32 (<.001) | .17 (<.001) | .12 (<.001) | -0.01 (.688) | .17 (<.001) | .13 (<.001) | 1 |  |  |  |  |  |  |
| 8. PGS ANX C | .25 (<.001) | .10 (<.001) | .10 (<.001) | .04 (.152) | .17(<.001) | .13 (<.001) | .13 (<.001) | 1 |  |  |  |  |  |
| 9. PGS MDD C | .55 (<.001) | .27 (<.001) | .19 (<.001) | .03 (.235) | .23 (<.001) | .25 (<.001) | .20 (<.001) | .16 (<.001) | 1 |  |  |  |  |
| 10. PGS DEP C | .78 (<.001) | .31 (<.001) | .16 (<.001) | .02 (.388) | .24 (<.001) | .25 (<.001) | .21 (<.001) | .16 (<.001) | .58 (<.001) | 1 |  |  |  |
| 11. PGS SUD C | .35 (<.001) | .29 (<.001) | .07 (.006) | -.06 (.022) | .19 (<.001) | .16 (<.001) | .15 (<.001) | .08 (.002) | .18 (<.001) | .24 (<.001) | 1 |  |  |
| 12. INT | .10 (<.001) | .08 (.002) | .10 (<.001) | -.01 (.704) | .01 (.749) | .01 (.635) | .04 (.117) | .01 (.608) | .06 (.023) | .09 (<.001) | .06 (.014) | 1 |  |
| 13. EXT | .12 (<.001) | .16 (<.001) | .08 (.001) | -.03 (.201) | .02 (.552) | .01 (.792) | .07 (.008) | -.03 (.202) | .05 (.067) | .10 (<.001) | .11 (<.001) | .54 (<.001) | 1 |

Note: p-PGS= Polygenic score for genomic p, C=Child, PGS= Polygenic scores, ADHD=Attention-Deficit/Hyperactivity Disorder, ASD= Autism Spectrum Disorder, OCD= Obsessive-Compulsive Disorder, SCZ= Schizophrenia, BD= Bipolar Disorder, PTSD= Post-Traumatic Stress Disorder, ANX= Anxiety, MDD= Major Depressive Disorder, DEP= broadly defined depression, SUD= Substance use disorder, INT=Internalising behaviours, EXT=Externalising behaviours.

Table S4. Model fit parameter for path analysis

|  | Externalising | Internalising |
| --- | --- | --- |
| RMSEA | 0.042 | 0.043 |
| SRMR | 0.047 | 0.048 |
| CFI | 0.954 | 0.940 |
| TLI | 0.944 | 0.926 |

Reference for threshold values indicating model fit: Good fit: RMSEA < 0.10 (Steiger, 1990); SRMR < 0.08; CFI > 0.95; TLI > 0.95 (Hu & Bentler, 1999). Acceptable fit: CFI > 0.90 and TLI > 0.90 (Hu & Bentler, 1999).


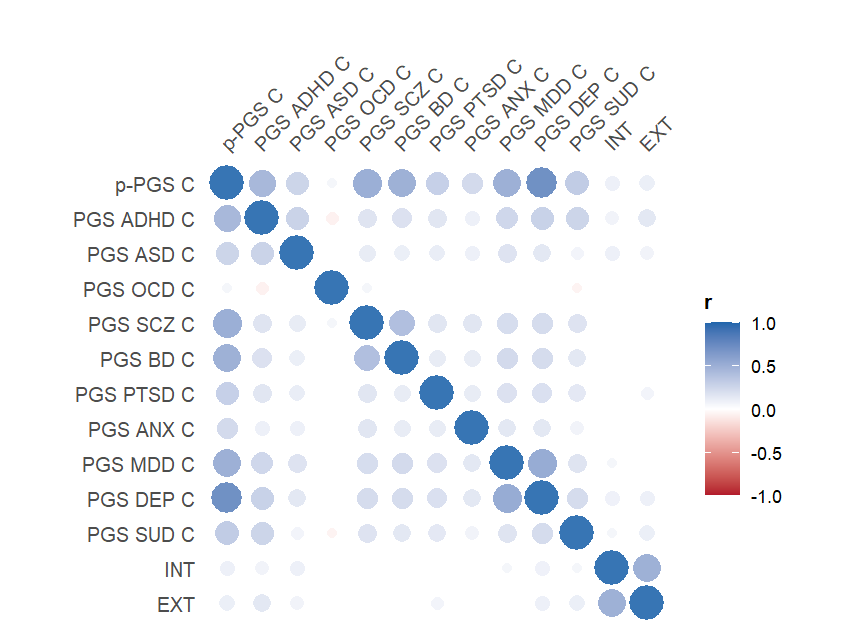


**Figure S1.** **Correlation matrix between p-PGS, disorder specific PGSs, and internalising and externalising behaviour.** Note: p-PGS= Polygenic score for genomic p, C=Child, PGS= Polygenic scores, ADHD=Attention-Deficit/Hyperactivity Disorder, ASD= Autism Spectrum Disorder, OCD= Obsessive-Compulsive Disorder, SCZ= Schizophrenia, BD= Bipolar Disorder, PTSD= Post-Traumatic Stress Disorder, ANX= Anxiety, MDD= Major Depressive Disorder, DEP= broadly defined depression, SUD= Substance use disorder, INT=Internalising behaviours, EXT=Externalising behaviours.


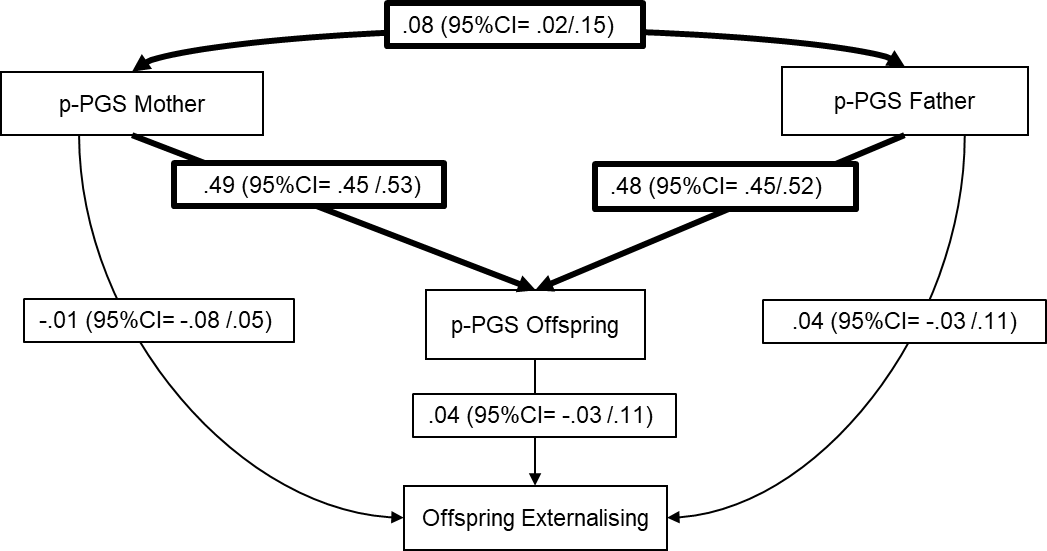


**Figure S2.** **Sensitivity analysis testing genetic nurture for** **externalising behaviour.** The path model accounts for the covariance between internalising and externalising outcomes (r = 0.55, p < 0.001).


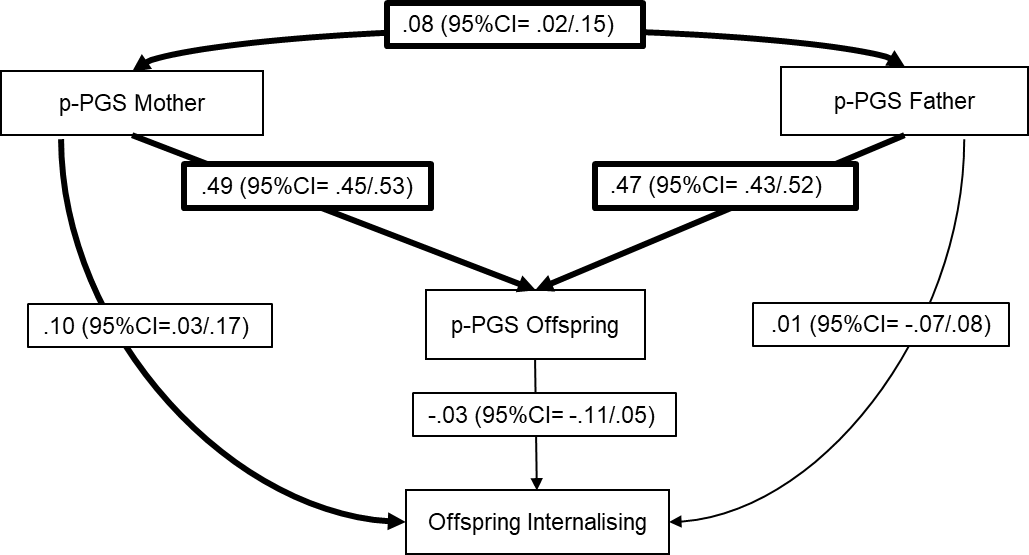


**Figure S3.** **Sensitivity analysis testing genetic nurture for internalising behaviour.** The path model accounts for the covariance between internalising and externalising outcomes (r = 0.56, p < 0.001).
